# PINC: A Tool for Non-Coding RNA Identification in Plants Based on an Automated Machine Learning Framework

**DOI:** 10.3390/ijms231911825

**Published:** 2022-10-05

**Authors:** Xiaodan Zhang, Xiaohu Zhou, Midi Wan, Jinxiang Xuan, Xiu Jin, Shaowen Li

**Affiliations:** 1 Anhui Province Key Laboratory of Smart Agricultural Technology and Equipment, Anhui Agricultural University, Hefei 230036, China; 2 College of Information and Computer Science, Anhui Agricultural University, Hefei 230036, China

**Keywords:** plant, ncRNA identification, AutoGluon, tool

## Abstract

There is evidence that non-coding RNAs play significant roles in the regulation of nutrient homeostasis, development, and stress responses in plants. Accurate identification of ncRNAs is the first step in determining their function. While a number of machine learning tools have been developed for ncRNA identification, no dedicated tool has been developed for ncRNA identification in plants. Here, an automated machine learning tool, PINC is presented to identify ncRNAs in plants using RNA sequences. First, we extracted 91 features from the sequence. Second, we combined the F-test and variance threshold for feature selection to find 10 features. The AutoGluon framework was used to train models for robust identification of non-coding RNAs from datasets constructed for four plant species. Last, these processes were combined into a tool, called PINC, for the identification of plant ncRNAs, which was validated on nine independent test sets, and the accuracy of PINC ranged from 92.74% to 96.42%. As compared with CPC2, CPAT, CPPred, and CNIT, PINC outperformed the other tools in at least five of the eight evaluation indicators. PINC is expected to contribute to identifying and annotating novel ncRNAs in plants.

## 1. Introduction

RNA is the template that codes for the proteins required to create cellular functions. RNA is structurally similar to DNA, but its function and chemical composition are fundamentally different. At a higher level, RNA is divided into two main groups: coding RNA that accounts for approximately 2% of all RNAs, and non-coding RNA (ncRNA) that accounts for the majority (>90%) of RNAs [1]. Non-coding RNA refers to all RNAs that are transcribed from DNA but do not code for proteins. Additionally, ncRNA can be categorized into two groups according to the size of the sequence: long non-coding RNAs (lncRNAs) with sequences >200 nucleotides and small non-coding RNAs (sncRNAs) with sequences shorter than 200 nucleotides [2]. In previous research, ncRNAs have frequently been referred to as “useless genes” or transcriptional “noise” [3,4]. In contrast, a growing number of experiments have demonstrated that ncRNAs play important biological roles in a variety of biological processes, including gene regulation/expression, gene silencing, RNA modification and processing, as well as multiple important roles in life activities [5,6,7]. Numerous plant-specific biological processes, including the regulation of plant nutrient homeostasis, development, and stress responses, have been linked to ncRNAs [8,9,10]. MiRNAs and trans-acting siRNAs, for instance, contribute to leaf senescence in *Arabidopsis*; miR164 and its target ORE1 control leaf senescence in *Arabidopsis*, and as miR164 expression declines, ORE1 expression eventually increases [11]. In addition, overexpression of miR398b has been shown to decrease the transcript levels of genes encoding superoxide dismutase (CSD1, CSD2, SODX, and CCSD), which resulted in the production of reactive oxygen species (ROS) and increased rice resistance to *Magnaporthe oryzae* [12,13]. In recent years, to facilitate subsequent analyses and research of transcripts, ncRNA identification has been one of the tasks that needs to be addressed. Numerous bioinformatics methods and experiments have been developed for ncRNA identification and to evaluate their functions [14,15]. Genomic SELEX, microarray analysis, and chemical RNA-Seq are the most commonly used experimental techniques [16]; however, they are costly and time-consuming. Therefore, bioinformatics may be a more effective means of addressing the biological aspects of the problem.

Kong et al. developed the Coding Potential Calculator (CPC) in 2007 [17]. The CPC selected a number of biologically significant features, including ORF quality, coverage, and integrity. These features were incorporated into a support vector machine for coding potential identity, but its performance was dependent on sequence comparisons. CPC was revised in 2017 with the release of CPC2 [18]. CPC2 is faster and more accurate than CPC, and, as an input to the SVM model, it uses ORF size and integrity, a Fickett score, and the isoelectric point extracted from the original RNA sequence. CPC2 is a relatively neutral tool, which makes it somewhat more applicable to transcriptomes of non-model organisms. CPAT, developed by Wang et al. in 2013, is a logistic-regression-model-based ncRNA identification tool that classifies ncRNAs and cRNAs based on features such as ORF size and coverage, Fickett score, and hexamer score [19]. CNCI was proposed by Liang et al. in 2013, and while it is also based on the same SVM classifier as CPC2, it uses different features, categorizing ncRNA and cRNA based on ANT features [20]. CNIT is an updated version of CNCI that was released in 2019. CNIT employs the more robust integrated machine model XGBoost for classification [21]. Tong et al. introduced CPPred in 2019 [22] as an SVM-based tool. This tool distinguishes between ncRNAs and coding RNAs using the same ORF features as CPC2, as well as the isoelectric point, stability index, gravity three peptide, hexamer score, CTD, and Fickett score features. A number of tools have been published that can distinguish between ncRNA and coding RNA; however, the tools have some limitations, for example, their application is mainly limited to vertebrates and mammals. In addition, these tools rarely consider using plants for model training. Most tools only use the model plant *Arabidopsis*, and rarely involve other non-model plants. Moreover, since ncRNAs of animals are mainly transcribed by polymerase II, while ncRNAs of plants are mainly transcribed by RNA polymerase II, IV, and V [23], and ncRNAs are characterized by low-level expression and cross-species conservation [24], these tools for ncRNA identification in animals cannot guarantee the reliability in plants. Therefore, it is necessary to construct a powerful tool for ncRNA identification in plants.

Automatic machine learning (AutoML) is the process of applying machine learning to real-world problems in an automated manner. Since 2013, frameworks have been developed that have been based on the AutoML concept. AutoWEKA was the first AutoML framework to emerge [25]; it automatically selected models and hyperparameters. Additionally, H2O [26] and TPOT [27] were created. H2O is a JAVA-based framework that supports multiple types of grid searches to identify the optimal parameters following the generation of an integrated model. At its core, TPOT is a tree-based process optimization tool based on a genetic algorithm. Today, more and more frameworks, such as AutoGluon [28] and AutoKeras [29], have been developed based on the concept of AutoML. These frameworks have also been applied to Alzheimer’s disease diagnosis [30], biomedical big data [31], and additional bioinformatics fields [32].

In this experiment, we developed PINC, an AutoML-based instrument for the identification of ncRNAs and cRNAs in plants. The AutoML framework does not require a great deal of effort and time to optimize the model; it simply accepts the processed data as an input, tunes and sets the framework’s parameters, and then outputs the model automatically. Our experimental results include a number of significant contributions: (1) By combining the F-test and variance threshold, 10 out of 91 features were identified as being able to strongly distinguish between ncRNAs and coding RNA in plants. (2) Using the AutoML framework, a neutral model for non-coding RNA identification was obtained. (3) We combined the two previous points and developed a tool called PINC for ncRNA identification. After comparing PINC with the CPC2, CPAT, CNIT, and CPPred identification tools on nine independent test sets to validate the performance of PINC, we discovered that PINC performed exceptionally well on these independent test sets. This suggests that PINC is a reliable method for ncRNA identification in plants. In addition, users can upload their data for identification, which facilitates the study of plants that have received less attention.

## 2. Results

### 2.1. Training Setup

**Figure 1:**
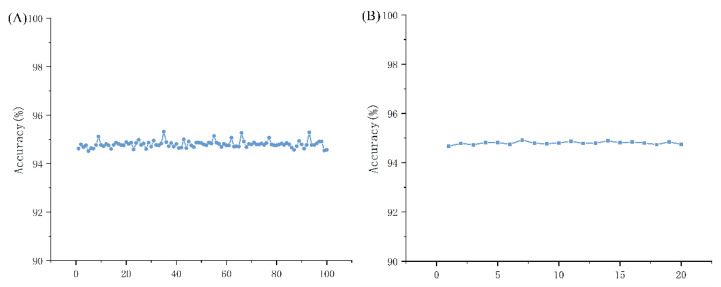
(**A**) Graph showing the accuracy of 100 experiments; (**B**) graph showing the average accuracy of every 5th experiment out of 100 experiments.

Once the features were selected, the models were tuned to find the best parameters, and the results were validated using a five-fold cross-validation procedure. A benchmark dataset of 4000 randomly selected data from each class was constructed for training and validation. Meanwhile, to ensure the validity of the experiments, we repeated the above experiments 100 times. As shown in Figure 1A, the highest accuracy of the 100 experiments was 95.32% and the lowest was 94.52%, mostly distributed between 94.6% and 94.9%, with very small fluctuations. For further proof, we averaged the accuracy of every fifth experiment, as shown in Figure 1B, and the curve fluctuates even less. This result shows that the randomly selected data is representative of the entire data set. Therefore, we took 4000 randomly selected data from each class as our baseline dataset.

### 2.2. Performance Comparison of the Feature Selection Methods

**Figure 2:**
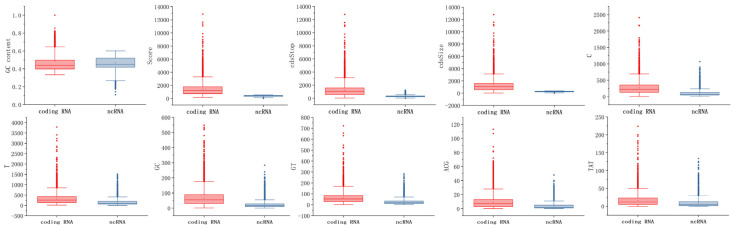
Differential distribution of ten features in coding RNAs and ncRNAs.

**Figure 3:**
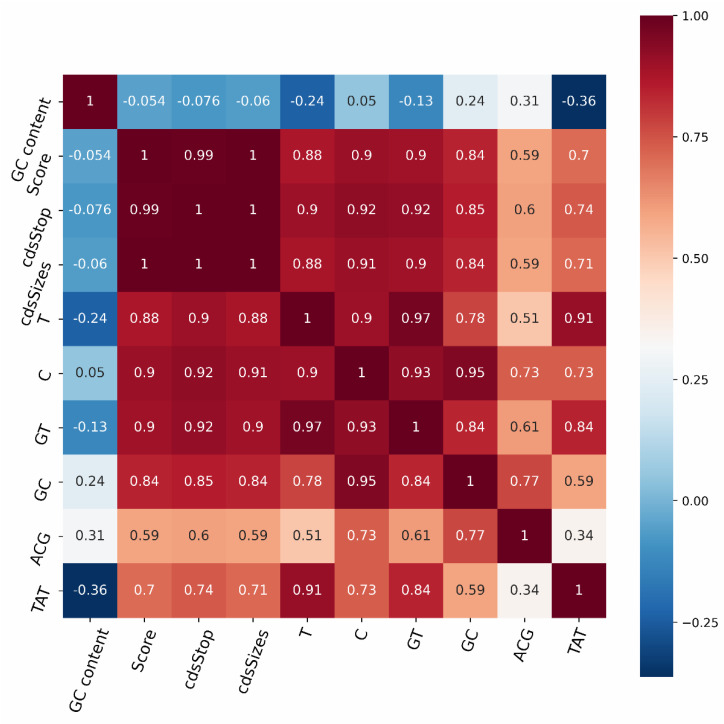
Correlation analysis chart of 10 features selected for the classification task.

In this research, 91 features were filtered using four feature selection methods: F-test, variance threshold, RF, and variance threshold combined with F-test (VT-F). These feature selection methods were compared in order to assess their usefulness. These feature selection methods use learning curves to continuously reduce the number of available features and to select the most appropriate features. The maximum validation set accuracy was 94.77 percent when the first 31 features were chosen using F-test filtering, and it was 94.29 percent when the first 25 features were chosen using variance threshold filtering. For VT-F, features below the mean were first filtered out using a variance threshold, and then the remaining features were filtered using the F-test, with a maximum accuracy of 95.25 percent when the first 10 features were selected. The evaluation of the three previously described feature selection methods was based on the AutoGluon model. For RF, the range of features was narrowed down based on the importance of the features, with the highest accuracy of 93.27 percent when the first 21 features were selected, and the 21 features were then fed into AutoGluon with an accuracy of 94.72 percent. In addition to accuracy, we compared SE, F1, MCC, and SPC performance metrics. Table 1 demonstrates that the method combining the F-test and variance threshold for feature selection outperformed the other methods, and the 10 features it selected were GC content, score, cdsStop, cdsSize, and T, C, GT, GC, ACG, and TAT frequencies. The experiments analyzed the distribution of ncRNAs and coding RNAs on the dataset for these 10 features, and based on Figure 2, it can be seen that they play a significant role in the identification of discriminatory power. In addition, we conducted a correlation analysis between the ten features selected for the classification task. Figure 3 showed that, GC content had a weak correlation with the other features. Score, cdsStop, and cdsSize showed a stronger correlation with the other features. T, C, GT, GC, ACG, and TAT frequencies had the strongest correlation with the other features.

### 2.3. Comparison of Models

Regarding the validation set, in this study, we compared the five-fold cross-validation results of four AutoML frameworks, AutoGluon, TPOT, H2O, and AutoKeras, to those of three conventional machine learning models, i.e., random forest, SVM, and Naive Bayes (Table 2). It is evident that, in general, conventional machine learning models are less effective than AutoML; three of the four automated machine learning frameworks produced more effective models than the random forest, the best performing conventional machine learning model. AutoGluon achieved the best results for five of the eight evaluation metrics within the AutoML framework: ACC, F1, MCC, NPV, and SE. H2O achieved the best results for AUC, while Autokeras achieved the best results for PPV and SPC. It is evident that the AutoGluon framework is more effective than the other frameworks, possibly because AutoGluon employs per-variable embedding, which improves quality via gradient flow, whereas the other frameworks merely apply the standard feed-forward architecture to hot-coded data. The Autokeras effect, which is based on NAS that combines multiple search strategies such as random search, grid search, etc., is only marginally weaker than the AutoGluon effect. The goal of NAS is to reduce human intervention and to allow the algorithm to design the neural network automatically, which consists of three key components: the search space, the search strategy, and the evaluation strategy. However, this process is typically very time-consuming. H2O had the highest AUC score, but its overall performance was comparable to that of conventional machine learning models and TPOT. H2O is a distributed machine learning platform based on the Java programming language, unlike other AutoML frameworks. TPOT was the least effective AutoML and the only framework with overall lower results than conventional machine learning models. This is likely due to the genetic algorithm employed by TPOT, which tends to converge on a locally optimal solution prematurely. Consequently, the comparison demonstrates that the models created by the AutoGluon framework are superior to those created by the other four automatic machine learning frameworks and the three conventional machine learning models.

### 2.4. Comparison Tools against Plant Datasets

**Figure 4:**
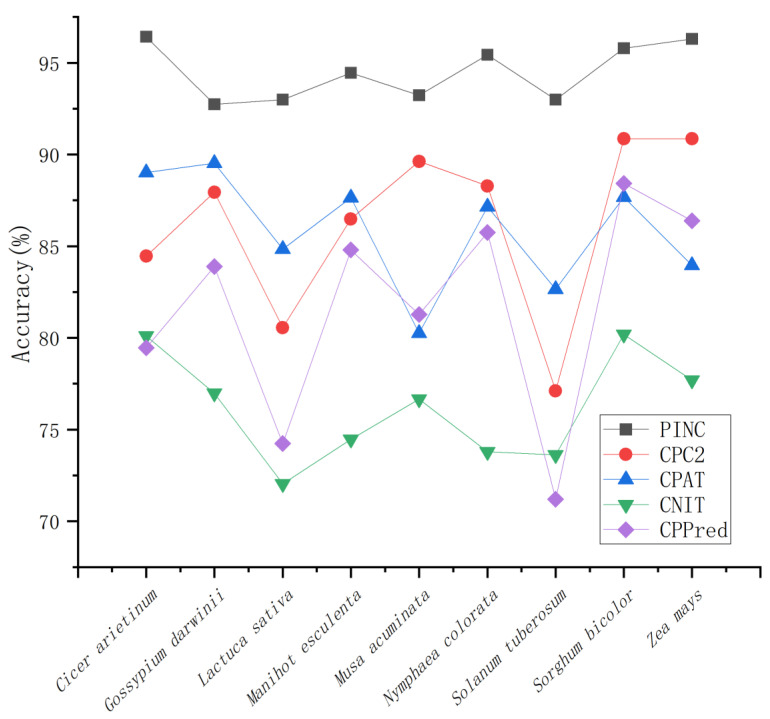
Comparing the identification accuracy of nine independent test sets across five tools.

**Figure 5:**
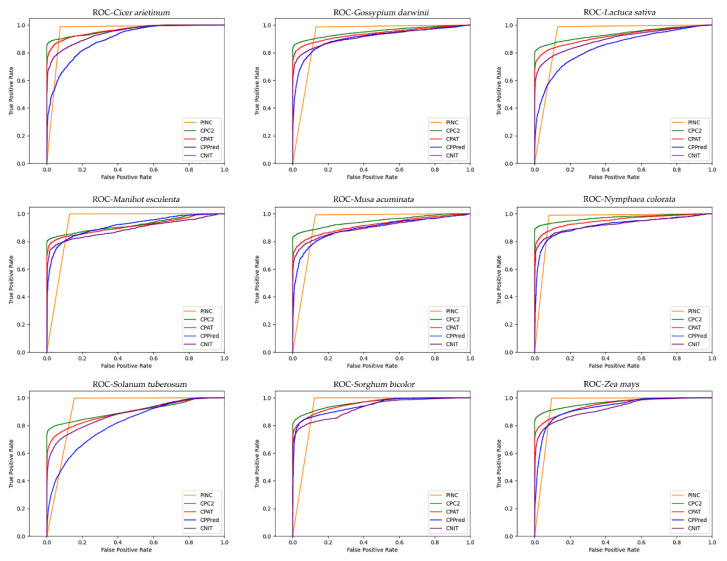
ROC curves for 5 tools on 9 plants.

**Figure 6:**
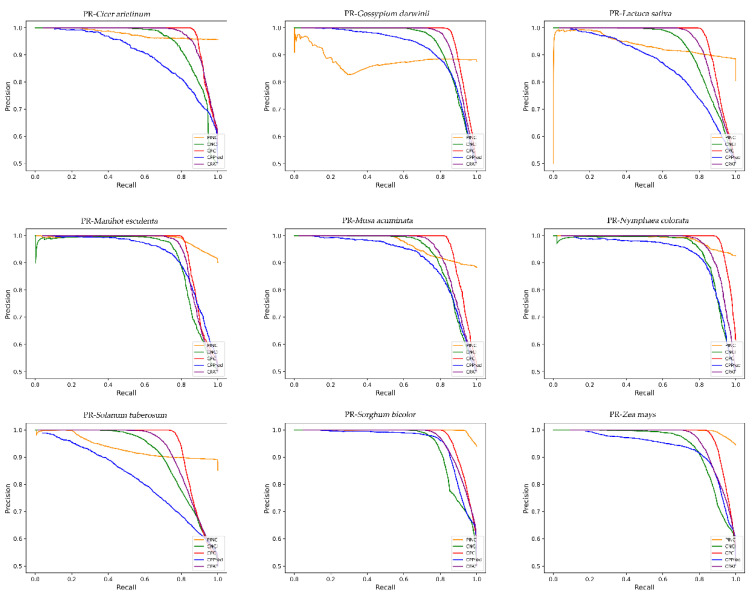
The PR curves obtained by PINC and four existing tools.

To evaluate the accuracy of PINC in ncRNA and coding RNA identification, we compared it to CPC2, CPAT, CNIT, and CPPred. We compared the identification accuracy for nine plant species from four databases, GreeNC, CANTATA, RNAcentral, and Phytozome, using five different tools. It is evident from the results shown in Figure 4 that our tool has the highest degree of precision for all nine plants. The large fluctuation of CPPred indicates that it has poor generalization performance, whereas the other three tools have some stability. However, it can be seen that the identification accuracy of PINC is greater than that of the other three tools, indicating that our tool performs the best among the different plant species. To compare the performances of these five tools further, we used eight metrics: sensitivity (SE), specificity (SPC), accuracy (ACC), F1-score, PPV, NPV, MCC, and AUC to evaluate and compare the five tools for these nine independent test sets (Table 3). We plotted the ROC curve (Figure 5); it can be seen that the ROC curve for PINC differs from the other tools. A true positive rate is rapidly achieved (1.0) at the cost of a relatively high false positive rate. Therefore, we have also plotted PR curves (Figure 6) to further illustrate the performance of PINC. The results showed that the PR curve of PINC did not fluctuate markedly and had a decreasing trend when the threshold was greater than 0.8. Meanwhile, PR curves illustrated that Precision and Recall values of five plants (*Cicer arietinum*, *Manihot esculenta*, *Nymphaea colorata*, *Sorghum bicolor*, and *Zea mays*) were higher than the other tools at the same threshold. All those results showed that PINC had the superior performance for distinguishing ncRNAs from coding RNAs. *Solanum tuberosum* outperformed the other tools in seven of the eight evaluation metrics and at least five of the remaining eight test sets, namely, SE ACC, F1, NPV, and MCC. The high Se score indicates that the probability of missing is small; therefore, PINC is the best choice for ncRNA identification. For the specificity SPC score, only one dataset was higher than the other tools, with four datasets performing best on CNIT and two datasets performing best on CPC2 and CPAT, respectively. However, the difference between the SPC of PINC and the SPC of the other tools was not large, and all tools had high performances above 86.99%. Among the five tools, PINC was the most effective for ncRNA identification in the nine plants. This indicates that our tool has a strong generalization to plants, which is crucial for non-model plants.

## 3. Discussion

In the field of bioinformatics, automated machine learning methods are now beginning to be implemented. In our experiments, we compared four automatic machine learning frameworks that are good matches for the more recently introduced frameworks and the older frameworks. For all the automatic machine learning frameworks, we used the same preprocessing methods to process the data as a raw input, then, we adjust the parameters of each framework in order to find the most suitable parameters, and finally we output the model. In general, we consider automatic machine learning frameworks to be black boxes and do not examine frame-specific methods for automatically optimizing parameters and integrating the model for direct output. Automated machine learning frameworks automatically optimize models, thereby reducing the time and effort devoted by researchers and, to a certain extent, allowing non-experts in machine learning to solve bioinformatics problems.

Utilizing high-quality features is one way to improve performance in machine learning. It is necessary to find features that are suitable for ncRNA identification in the study because providing or discovering good features is one of the most important tasks in machine learning. We extracted k-mer frequency features, coding sequence features, and other features during our experiments. Despite the fact that traditional k-mer features have been used in a variety of studies, such as gene identification [33], subcellular localization [34], and sequence analysis, it has been demonstrated that the k-mer frequency is highly effective at detecting ncRNAs [35]. Many tools have also used features related to coding sequences and some other features [36]. Ninety-one extracted features were filtered using our feature selection method; the filtered features successfully identified ncRNAs and it was the most precise tool, to date, for ncRNA identification in all plant species.

For ncRNA identification, there are additional factors to consider, such as the trade-off between sensitivity and specificity. At present, the number of ncRNAs is small as compared with the number of coding RNAs identified. To prevent ncRNAs from being missed, high sensitivity is important. Currently, CPAT, CNCI, CPPred, and CPC2 are less sensitive and focus more on identifying coding RNAs, but this requires an additional step to screen for non-coding RNAs. In contrast, the high sensitivity of PINC reduces the necessity for additional filtering processes. Moreover, PINC demonstrated a higher rate of accuracy than any other tool among the nine plants evaluated. Although some tools for non-coding RNA identification have reached over 85 percent accuracy, increased accuracy is not meaningless, as large amounts of data have become available due to advances in sequencing technology, and it is possible that for every one percent increase, hundreds of additional correct RNAs can be identified. Here, PINC achieves a high degree of ncRNA identification precision. This may be because the model in PINC adopts the stacking strategy, while other tools use single models such as SVM, logistic regression, and xgboost. For a long time, the performance of combining the predicted results of multiple models has been better than that of a single model, and the variance has been significantly reduced [37]. In the experiment, we selected the default basic model in the AutoGluon framework. Here, the basic model is trained separately, and then the prediction of the basic model is used as a feature to train the stacked model. Stacked models can improve the shortcomings of a single-model prediction and can take advantage of their interactions to improve the prediction ability [38]. In addition, it can be seen from the feature level distribution map described earlier that these features also have strong discrimination ability.

In addition, we plan to continue research in two areas: first, deep learning, which can automatically extract features, reduce the time required to extract features, and can improve the accuracy of cross-species recognition. In contrast, we should consider machine learning techniques to gain a deeper understanding of these RNA types and to investigate their biological significance. In addition, for plants, only a handful of ncRNA functions have been identified; once these functions are identified, new mechanisms can be explored and new features can be added to PINC to improve our tool further.

## 4. Materials and Methods

**Figure 7:**
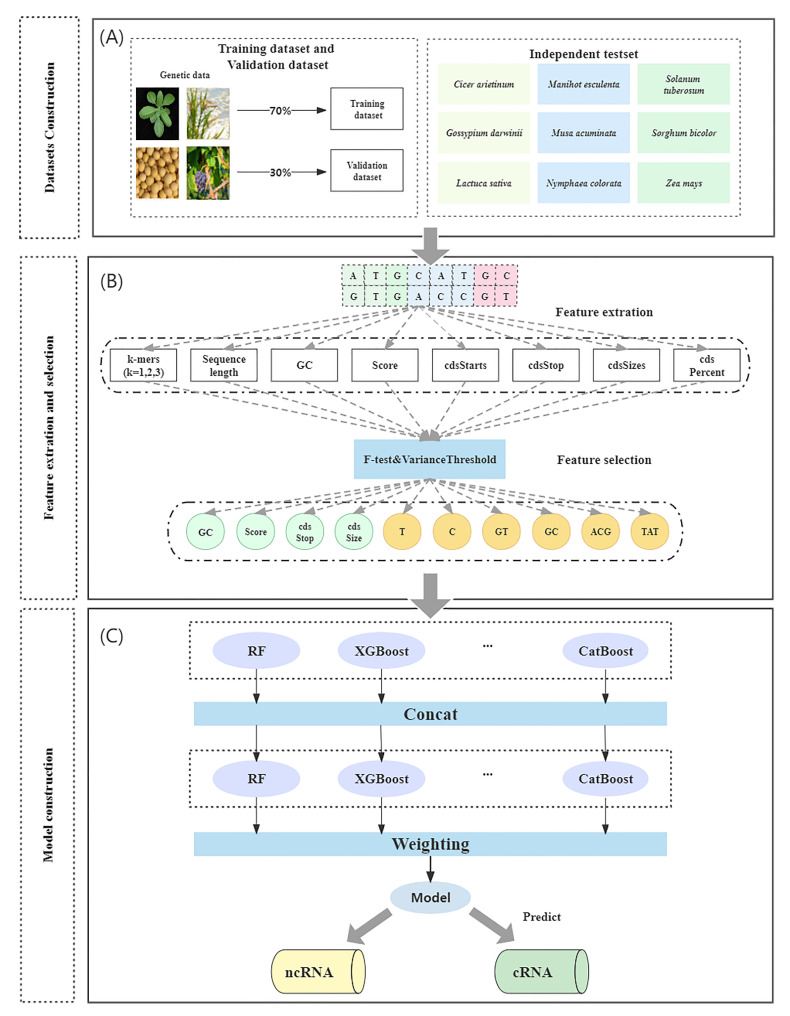
Overall workflow: (**A**) dataset construction: a dataset was constructed using four species of plants together for training and validation, and nine independent test sets were constructed for testing; (**B**) feature extraction: features were extracted from the original sequence species and redundant features were filtered out using feature selection methods; (**C**) model construction: a stacking strategy was used to integrate multiple models.

Figure 7 depicts the tool’s overall workflow, which consists of three steps: (1) dataset construction, (2) feature extraction and selection, and (3) model construction.

To create the dataset, RNA sequences were obtained from the GreeNC, CANTATA, RNAcentral, and Phytozome databases. Secondly, feature selection methods were used to extract and filter features. Finally, machine learning models were compared to determine the most effective model for ncRNA identification.

### 4.1. Dataset Construction

**Figure 8:**
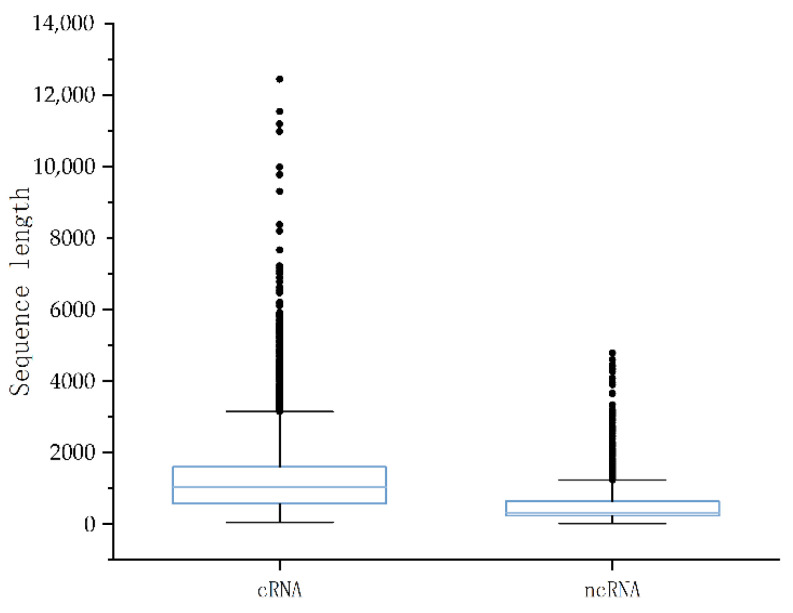
Distribution of positive and negative sample lengths in the benchmark dataset.

To construct the experimental dataset, we considered two factors. On the one hand, the diversity of plants and the abundance of annotation data were taken into consideration. On the other hand, considering the balance of the data, we chose four plants as our training and validation datasets (Table 4), which included two model plants, i.e., *Arabidopsis thaliana* and *Oryza sativa*, in addition to two non-model plants, i.e., *Glycine max* and *Vitis vinifera*. We used ncRNAs as the positive sample data and coding RNAs as the negative sample data in the dataset. Negative samples were obtained from Phytozome.v13 [39]. Positive samples were obtained from three public databases, including GreeNC [40], CANTATA [41], and RNAcentral [42]. For all data, first, we used cd-hit-est-2D in the CD-hit tool [43] to eliminate redundant sequences between the test and training sets at a threshold of 80% [22,35,44,45]. Second, in order to balance the datasets, random selections of 4000 data were made for each plant, of which 2000 were positive samples and 2000 were negative samples. The positive sample data consisted of 1800 lncRNAs and 200 sncRNAs, and the negative sample data consisted of 2000 mRNAs (Table 5) [18,46]. Thus, the baseline dataset consisted of a total of 16,000 protein sequences from four plants. Meanwhile, we analyzed the length distribution of the positive and negative datasets, as shown in Figure 8. The median length of the coding RNAs data was 1029 and the data were mostly concentrated in the range of 0–2000. The ncRNA data had a median length of 321 and the data were mostly concentrated in the range of 0–1000. Finally, we proportionally divided the dataset into 70% training data and 30% validation data. Additionally, nine independent test sets were created for nine plants. (Table 6): *Cicer arietinum*, *Gossypium darwinii*, *Lactuca sativa*, *Manihot esculenta*, *Musa acuminata*, *Nymphaea colorata*, *Solanum tuberosum*, *Sorghum bicolor*, and *Zea mays.* To eliminate redundant sequences, the data for these nine independent test sets were taken from the four databases mentioned above and filtered at a threshold of 80%.

### 4.2. Feature Extraction and Selection

This experiment initially extracted 91 features (Table 7). The 86 features of k-mer frequency, sequence length, and GC content were obtained using the Python script program (https://github.com/midisec/PINC, accessed on 22 August 2022); the five features of Score and CDS were obtained using the UCSC Genome txCdsPredict program in the browser (http://hgdown-load.soe.ucsc.edu/admin/jksrc.zip, accessed on 11 November 2014) [47]. These features can be classified into three categories: k-mer frequency features, CDS-related features, and other features. The k-mer frequency describes all possible frequencies for the presence of k nucleotides in a sequence, based on methods that have initially been implemented in whole genome shotgun assemblers. When k = 1, each nucleotide can contain a maximum of four A, C, G, or T. When k equals 2, the calculation involves the dinucleotide frequency (i.e., AA, AT, AG, AC, …, TT) and a total of $4^{2}$= 16 species. When k = 3, the calculated three-nucleotide frequencies (i.e., AAA, AAT, AAG, AAC, …, TTT) are computed for a total of $4^{3}$= 64 species. By combining 1–3-mer frequencies for a total of 84 features, k-mer frequencies can capture rich statistical information about negative profiles in plant genomes, according to some research [48]. CDS is the result of encoded proteins that are interchangeable with ORF in some ways, but differ slightly [49]. The features Score, cdsStarts, cdsStop, cdsSize, and cdsPercent comprise the second major category of features. Score is the predicted protein score; if it is >800, there is a 90% chance that it is a protein, and if it is >1000, it is virtually certain that it is a protein. cdsStop is the end of the coding region in the transcript, cdsSize is cdsStop minus cdsStart, and cdsPercent is the ratio of cdsSize to the total sequence length. Other features include sequence length and GC content, which are widely used for ncRNA identification. Sequence length indicates the total length of the sequence. GC content is the ratio of guanine and cytosine to the other four DNA bases.

There may be redundant features among the 91 features listed above; therefore, we employed feature selection to filter them. For the feature selection method, redundant features were filtered out using a combination of variance threshold filtering and the F-test. Variance threshold filtering is used to filter features based on their own variance. The smaller a feature’s variance, the less significant its variation, and these insignificant features are eliminated. F-test is a method to determine the relationship between each feature and label. The GC content, Score, cdsStop, cdsSize, and T, C, GT, GC, ACG, and TAT frequencies were among the 91 features identified by this combined feature selection method. Finally, these 10 features were used as the model input.

### 4.3. Model Construction

Machine learning (ML) is currently utilized in a variety of fields to solve numerous difficult problems. Nevertheless, model construction for machine learning requires human intervention. Manual intervention is required in the feature extraction, model selection, and parameter adjustment processes, which require professionals to optimize and can waste a significant amount of time and resources if errors occur. To reduce these repetitive development costs, the concept of automating the entire machine learning process, automatic machine learning, has been conceived (AutoML). The definition of AutoML is that it is a combination of automation and ML [50]. From an automation standpoint, AutoML can be viewed as the design of a framework to automate the entire machine learning process, allowing models to automatically learn the correct parameters and configurations without manual intervention. From the standpoint of machine learning, AutoML is a system that is highly capable of learning and generalizing given data and tasks. Recent research on AutoML has focused on the neural network architecture search (NAS) method, which employs a search strategy to test and evaluate a large number of architectures in a search space, and then selects the one that best meets the objectives of a given problem by maximizing the adaptation function. However, the NAS faces two obstacles to the method: first, the amount of computation is excessive, resulting in increased resource consumption. Second, instability may vary each time and the search structure is altered, resulting in varying precision. In our experiments, we compared four automatic machine learning frameworks, AutoGluon, H2O, TPOT, and Autokeras, with three conventional machine learning models, SVM, RF, and Naive Bayes. We determined that AutoGluon was the superior framework, and therefore it was used as the classifier. AutoGluon contains 26 base models including random forest, XGBoost, and a neural network, and in our experiments, we used all the base models for training the model [51]. AutoGluon is an open-source machine learning training framework for tabular data. It is a framework that attempts to avoid a hyperparametric search as much as possible, training multiple models concurrently and weighting them using a multi-layer stacking strategy to obtain the final output.

### 4.4. Performance Evaluation

Several widely used performance metrics were evaluated in the experiments, including sensitivity (SE), specificity (SPC), accuracy (ACC), F1-score, positive predictive value (PPV), negative predictive value (NPV), and the Matthews correlation coefficient (MCC). To evaluate the performance of the classifier numerically and visually, the area under the curve (AUC) and ROC curves were also used. These definitions are as follows:


$$Sensitivity(SE)=\frac{TP}{TP+FN}\;Specificity(SPC)=\frac{TN}{TN+FP}\;Accuracy(ACC)=\frac{TP+TN}{TP+FN+FP+TN}\;F1=\frac{2\times TP}{2\times TP+FP+FN}\;PPV=\frac{TP}{TP+FP}\;NPV=\frac{TN}{TN+FN}\;MCC=\frac{TP\times TN-FP\times FN}{\sqrt{\left(TP+FN)\times (TP+FP)\times (TN+FP)\times (TN+FN\right)}}$$

TP represents true positives, the number of correctly identified positive samples, while FN, TN, and FP represent false negatives, true negatives, and false positives, the number of incorrectly identified positive samples, correctly identified negative samples, and incorrectly identified negative samples, respectively.

## 5. Conclusions

Various tools have been developed to distinguish between ncRNAs and coding RNAs, the majority of which have used scientific computational methods to differentiate sequences and to accelerate the annotation of various human genes. In addition to nucleotides with high discriminatory power in 1–3-mer, we also extracted other features such as the sequence’s definition, composition, and function. Moreover, we combined F-test and variance threshold filtering and found that the combined method was superior to the individual methods of F-test and variance threshold filtering. A number of automated machine learning and traditional machine learning frameworks were also used for modeling, in which the validation set was carefully evaluated and analyzed, including the use of cross-validation on the validation set available, with AutoGluon performing the best. Then, we compiled these into a tool called PINC and compared it to nine other tools on nine test sets, demonstrating that PINC performed better than other tools on all of these species. For user convenience, a user-friendly web (http://www.pncrna.com/, accessed on 22 August 2022) has been developed, where the output can be obtained simply by entering a FASTA sequence or file. Overall, PINC has excellent predictive properties, permits cross-species plant identification, and is a practical and user-friendly tool.

## Figures and Tables

**Table 1:** Comparing the performance of different feature selection methods.

	SE	SPC	ACC	MCC	F1
F-test	99.49	90.18	94.77	89.95	94.93
VT	99.49	89.24	94.29	89.08	94.49
VT-F	**99.79**	90.7	**95.27**	**90.91**	**95.49**
RF	90.35	**96.52**	93.27	86.76	93.39
RF-AutoGluon	99.45	90.14	94.72	89.87	94.89

**Table 2:** Performance comparisons among five automated machine learning frameworks and three conventional machine learning models.

Model	ACC (%)	F1 (%)	AUC (%)	MCC (%)	NPV (%)	PPV (%)	SE (%)	SPC (%)
AutoGluon	**95.25**	**95.49**	95.25	**90.91**	**99.76**	91.55	**99.79**	90.70
Naive Bayes	86.97	87.96	86.93	74.83	79.22	94.65	82.16	93.63
SVM	53.14	13.67	53.37	16.93	99.33	0.07	91.65	51.51
RFC	92.10	92.26	92.09	84.26	90.45	93.73	90.86	93.47
H2O	92.98	93.38	**96.80**	86.60	86.98	98.98	88.38	98.84
TPOT	86.14	86.19	86.15	72.29	86.18	86.10	86.28	86.00
Autokeras	93.70	94.06	94.57	87.95	88.10	**99.25**	89.39	**99.15**

**Table 3:** Nine plants’ performance indicators were compared using five tools.

Species	Tool	SE (%)	SPC (%)	ACC (%)	F1 (%)	PPV (%)	NPV (%)	MCC (%)	AUC (%)
*Cicer arietinum*	PINC	**98.76**	92.46	**96.42**	**97.20**	**95.70**	**97.72**	**92.34**	95.61
	CPC2	76.01	92.91	84.45	83.04	91.50	79.42	69.92	**96.50**
	CPAT	89.27	88.75	89.01	89.05	88.84	89.18	78.02	96.26
	CNIT	65.65	**94.67**	80.10	76.81	92.54	73.23	62.99	94.36
	CPPred	71.24	87.70	79.46	77.65	85.32	75.24	59.75	89.72
*Gossypium darwinii*	PINC	**98.61**	86.84	**92.74**	**93.16**	88.29	**98.41**	**86.08**	92.72
	CPC2	85.25	90.62	87.94	87.60	**90.09**	86.00	75.99	**95.23**
	CPAT	95.03	84.02	89.53	90.07	85.61	94.42	79.54	93.65
	CNIT	63.23	**90.73**	76.98	73.31	87.21	71.16	56.13	90.67
	CPPred	80.78	86.59	83.89	82.36	84.00	83.80	67.59	91.66
*Lactuca sativa*	PINC	**98.80**	87.00	**92.99**	**93.47**	**88.68**	**98.60**	**86.54**	92.90
	CPC2	70.14	90.96	80.56	78.30	88.58	75.30	62.49	**93.56**
	CPAT	87.24	82.50	84.84	85.03	82.94	86.90	69.79	92.00
	CNIT	51.95	**92.24**	72.03	65.08	87.09	65.59	48.26	89.79
	CPPred	64.39	84.06	74.23	71.42	80.16	70.24	49.42	84.12
*Manihot esculenta*	PINC	**99.82**	**87.12**	**94.45**	**95.40**	**91.36**	**99.72**	**88.99**	**93.47**
	CPC2	87.82	85.14	86.48	86.66	85.53	87.48	72.99	92.15
	CPAT	93.55	81.73	87.64	88.33	83.66	92.68	75.81	91.13
	CNIT	62.73	86.18	74.46	71.06	81.94	69.82	50.33	91.30
	CPPred	87.5	80.08	84.79	85.19	83.00	86.78	69.68	88.97
*Musa acuminata*	PINC	**99.22**	87.22	**93.23**	**93.61**	88.61	**99.11**	**87.08**	93.22
	CPC2	90.12	**88.99**	89.62	90.6	**91.09**	87.83	79.02	**94.71**
	CPAT	71.69	88.82	80.25	78.41	86.54	75.79	61.42	91.84
	CNIT	65.08	88.28	76.66	73.63	84.77	71.6	54.85	90.14
	CPPred	76.44	86.1	81.27	80.33	84.64	78.48	62.84	89.24
*Nymphaea colorata*	PINC	**98.82**	91.94	**95.44**	**95.66**	92.69	**98.70**	**91.08**	95.38
	CPC2	82.69	**93.85**	88.28	87.57	**93.06**	84.45	77.03	**97.08**
	CPAT	82.9	91.39	87.14	86.57	90.59	84.24	74.56	95.10
	CNIT	55.21	92.38	73.79	67.81	87.88	67.34	51.27	92.24
	CPPred	84.89	86.59	85.74	85.62	86.36	85.14	71.49	91.56
*Solanum tuberosum*	PINC	**99.73**	84.53	**92.99**	**94.06**	**89.00**	**99.60**	**86.41**	**92.13**
	CPC2	67.23	86.99	77.11	74.60	83.79	72.63	55.31	90.61
	CPAT	86.69	78.61	82.65	83.31	80.18	85.54	65.51	89.47
	CNIT	58.76	**88.49**	73.62	69.02	83.63	68.20	49.49	88.12
	CPPred	60.64	81.75	71.20	67.80	76.87	67.50	43.38	81.24
*Sorghum bicolor*	PINC	**99.9**	87.69	**95.79**	**96.92**	**94.11**	**99.79**	**90.69**	93.79
	CPC2	94.38	87.32	90.85	91.16	88.16	93.95	81.91	**96.42**
	CPAT	86.65	**88.71**	87.68	87.55	88.46	86.93	75.38	95.71
	CNIT	75.04	85.34	80.19	79.10	83.63	77.40	60.71	92.89
	CPPred	91.81	85.04	88.42	88.80	85.98	91.21	77.02	94.28
*Zea mays*	PINC	**99.71**	90.38	**96.30**	**97.16**	**94.74**	**99.45**	**92.12**	95.04
	CPC2	90.81	90.88	90.85	90.84	90.87	90.82	81.70	**96.63**
	CPAT	76.52	**91.37**	83.96	82.64	89.82	79.63	68.67	95.07
	CNIT	65.24	90.10	77.69	74.49	86.78	72.24	57.15	92.50
	CPPred	84.83	87.92	86.38	86.16	87.54	85.29	72.80	93.05

**Table 4:** Training set data for the model.

Species	Noncoding		Coding	
	Total	Used	Total	Used
*Arabidopsis thaliana*	45,910	2000	27,416	2000
*Glycine max*	8599	2000	71,358	2000
*Oryza sativa*	11,338	2000	42,189	2000
*Vitis vinifera*	4301	2000	55,564	2000
Total	70,148	8000	196,527	8000

**Table 5:** Detailed description of the training set data.

		Size
Non-coding RNAs	Long ncRNAs	1800
	Small ncRNAs	200
Coding RNAs	mRNAs	2000
Overall		4000

**Table 6:** Plant dataset for testing.

Species	Coding	Noncoding	Total
*Cicer arietinum*	2099	2099	4198
*Gossypium darwinii*	5622	5622	11,244
*Lactuca sativa*	4682	4682	9364
*Manihot esculenta*	2808	2808	5616
*Musa acuminata*	2059	2063	4122
*Nymphaea colorata*	1708	1708	3416
*Solanum tuberosum*	8282	8282	16,564
*Sorghum bicolor*	8657	8657	17,314
*Zea mays*	7406	7406	14,812

**Table 7:** All features considered in this paper.

Features	Description	Source
k-mer frequency	1–3 k-mer = 84	PINC
	1 nt = 4 features; 2 nt = 16 features	
	3 nt = 64 features	
Score	Values >800 are likely to be a protein, >1000 must be protein	txCdsPredict
cdsStarts	NT position of CDS starts from the transcript and is based on zero	txCdsPredict
cdsStop	nt position for the CDS end	txCdsPredict
cdsSizes	cdsStop-cdsStart	txCdsPredict
cdsPercent	(cdsStop + cdsStart)/total nt sequence size	txCdsPredict
Sequence length	Total nucleotide length of the sequence	PINC
GC content	$\frac{C+G}{A+C+G+T}$	PINC

## Data Availability

The data presented in this study are available at https://github.com/midisec/PINC, accessed on 22 August 2022.
